# The Prognostic Significance of Apoptosis-Related Biological Markers in Chinese Gastric Cancer Patients

**DOI:** 10.1371/journal.pone.0029670

**Published:** 2011-12-28

**Authors:** Xiaowen Liu, Hong Cai, Hua Huang, Ziwen Long, Yingqiang Shi, Yanong Wang

**Affiliations:** 1 Department of Gastric Cancer and Soft Tissue Sarcoma, Fudan University Shanghai Cancer Center, Shanghai, China; 2 Department of Oncology, Shanghai Medical College, Fudan University, Shanghai, China; National Cancer Center, Japan

## Abstract

**Background and Objective:**

*The* prognosis varied among the patients with the same stage, therefore there was a need for new prognostic and predictive factors. The aim of this study was to evaluate the relationship of apoptosis-related biological markers such as p53, bcl-2, bax, and c-myc, and clinicopathological features and their prognostic value.

**Methods:**

From 1996 to 2007, 4426 patients had undergone curative D2 gastrectomy for gastric cancer at Fudan University Shanghai Cancer Center. Among 501 patients, the expression levels of p53, bcl-2, bax, and c-myc were examined by immunohistochemistry. The prognostic value of biological markers and the correlation between biological markers and other clinicopathological factors were investigated.

**Results:**

There were 339 males and 162 females with a mean age of 57. The percentages of positive expression of p53, bcl-2, bax, and c-myc were 65%, 22%, 43%, and 58%, respectively. There was a strong correlation between p53, bax, and c-myc expression (*P* = 0.00). There was significant association between bcl-2, and bax expression (*P*<0.05). p53 expression correlated with histological grade (*P* = 0.01); bcl-2 expression with pathological stage (*P* = 0.00); bax expression with male (*P* = 0.02), histological grade (*P* = 0.01), Borrmann type (*P* = 0.01), tumor location (*P* = 0.00), lymph node metastasis (*P* = 0.03), and pathological stage (*P* = 0.03); c-myc expression with Borrmann type (*P* = 0.00). bcl-2 expression was related with good survival in univariate analysis (*P* = 0.01). Multivariate analysis showed that bcl-2 expression and pathological stage were defined as independent prognostic factors. There were significant differences of overall 5-year survival rates according to bcl-2 expression or not in stage IIB (*P* = 0.03).

**Conclusion:**

The expression of bcl-2 was an independent prognostic factor for patients with gastric cancer; it might be a candidate for the gastric cancer staging system.

## Introduction

Although the incidence of gastric cancer has been substantially declining for several decades, it was still the fourth most common cancer and the second most frequent cause of cancer death [1], [2]. It was very important to predict precisely the risk of poor prognosis in order to maximize the therapeutic effect and to minimize the adverse effects in the treatment of cancer patients. Among the prognostic factors now available for gastric cancer, the most precise and important prognostic factor was the UICC TNM stage. As a result of the variability of prognosis within the same stage of gastric cancer, it was impossible to precisely predict the prognosis through the conventional staging procedures. Therefore, it was essential to search for the other specific factors to identify subgroups of patients with more aggressive course of the disease. Recently, some studies have shown that other pathological factors, such as the number of positive lymph nodes, the presence of extracapsular lymph node involvement, and tumor size had additional prognostic value [3]–[5]. Given that some biological markers, such as oncogenes, tumor-suppressor genes, cell-cycle regulators, and DNA repair genes, were related to tumor genesis, growth, invasion and metastasis, many scientists were dedicated to searching for the new prognostic factors with these molecular markers. Despite of the fact that a few of studies have evaluated the prognostic significance of the markers, such as p53, bcl-2, bax, and c-myc in gastric cancer, the results were controversial [6]–[11]. Moreover, these previous studies were conducted to research the additional prognostic value of the markers to the 6th UICC TNM stage, it was urgent to elucidate the relationship between these makers and the 7th UICC TNM stage. At last, there were no studies evaluating the expression of these molecular prognostic significance in gastric cancer patients in China. Therefore, we evaluated the expression of biological markers such as p53, bcl-2, bax, and c-myc in this study.

p53 proteins were the products of tumor-suppressor genes, which acted by modulating cell proliferation via control of the G1 arrest checkpoint of the cell cycle [12], [13]. bcl-2 and bax belonged to the bcl-2 family, and the latter was one of the most relevant classes of apoptosis regulatory molecules. The bcl-2 family were classified into two subfamilies: anti-apoptosis proteins (bcl-2, bcl-XL, bcl-w, bfl-1, brag-1, and mcl-1) and pro-apoptosis proteins (bax, bak, bcl-Xs, bad, bid, and hrk) [14]. It has been shown that the bax to bcl-2 could affect the relative sensitivity or resistance of cells to apoptotic simuli [15]. c-myc protein was a type of transcription factor. A previous study showed the c-myc expression was linked with cell proliferation [16].

In this study, we evaluated the expression of biological markers such as p53, bcl-2, bax, and c-myc, correlated their expression to clinicopathological characteristics and evaluated their prognostic value.

## Materials and Methods

### Patients

During January 1996 and December 2007, 4426 patients with histologically confirmed primary gastric adenocarcinoma underwent curative D2 gastrectomy at the Department of Gastric Cancer and Soft Tissue Sarcoma in Fudan University Shanghai Cancer Center. Out of these patients, 501 cases were selected, as their specimens were stained immunohistochemically after operation with routine histological examination. Data were retrieved from patients' operative and pathological reports, and follow-up data were obtained by phone, letter, and the outpatient clinical database. This study comprised 339 men and 162 women aged from 22 to 82 years old, mean age was 57 years. Staging was performed according to the UICC TNM Staging Classification for Carcinoma of the Stomach (Seventh Edition, 2010). A follow-up of all patients was carried out according to our standard protocol (every three months for at least 2 years, every six months for the next 3 years, and after 5 years every 12 months for life). The check-up items included physical examination, tumor-marker examination, ultrasound, chest radiography, computed tomographic scan, and endoscopic examination. The median follow-up time was 44.6 months at the time of analysis. The written informed consent had been obtained from all the patients, and this study was approved by the Ethical Committee of Shanghai Cancer Center of Fudan University. The study was retrospective.

### Immunohistochemical staining

p53, bcl-2, bax, and c-myc were detected by immunohistochemical method. All primary antibodies and mouse monoclonal antibodies were purchased from Dako (Hamburg. Germany). Immunohistochemical staining was performed by the enhance labeled polymer system (ELPS) method, with 10% formalin-fixed, paraffin-embedded materials. Serial 5 µm paraffin sections were deparaffinised with xylene and hydrated with ehanol/deionized water. Sections were then incubated with methanol/0.3% H_2_O_2_ for 10 min and blocked with non-specific staining blocking reagent. After overnight incubation at 4°C with anti-p53, bcl-2, bax, and c-myc antibody (diluted 1∶100, 1∶100, 1∶100, 1∶100, respectively), sections were treated according to standard immunoperoxidase methods using a streptavidin biotin peroxidise complex kit (LSAB+Kit/HRP; Dako). The peroxidise reaction was then developed with diaminobenzidine (Dako). Negative control sections were subjected to the same procedure except that the first antibody was replaced by PBS. No positive staining was observed in the controls. The position of staining was predominantly in cell cytoplasm for bax and c-myc, cytoplasm and cytomembrane for bcl-2, nucleus for p53.

### Immunohistochemical staining scoring

All the slices were evaluated by two pathologists without knowledge of clinical outcome. The percentage of immunoreactive cells and staining intensities were evaluated in each sample. The percentage of immunoreactive cells was graded on a scale of 0 to 4: no staining is scored as 0, 1–10% of cells stained scored as 1, 11–50% as 2, 51–80% as 3, and 81–100% as 4. The staining intensities was graded from 0 to 3: 0 is defined as negative, 1 as weak, 2 as moderated, and 3 as strong, respectively. The raw data were converted to the immunohistochemical score (IHS) by multiplying the quantity and intensity scores. An IHS score of 9–12 was considered as strong immunoreactivity (+++), 5–8 as moderate (++), 1–4 as weak (+), and 0 as negative (−). On the final analysis, the cases had a score of less than 1 were considered as negative, and ≥1 was regarded as positive.

### Statistical analysis

The association between clinicopathological factors and biological markers was evaluated by Chi-square test. Five-year survival rate was calculated by Kaplan-Meier method, and differences between survival curves were examined with log-rank test. All the significant variables observed in univariate analysis were included into the multivariate survival analysis using Cox proportional hazards model. The accepted level of significance was *P*<0.05. Statistical analyses and graphics were performed using the SPSS 13.0 statistical package (SPSS, Inc., Chicago, IL).

## Results

### Clinicopathological characteristics

There were 339 males and 162 females (2.09:1) with a mean age of 57 years. There was 57 (11.4%) early gastric cancers and 444 (88.6%) advanced gastric cancers. According to histological type, well-differentiated tumors were observed in 9 (1.8%) patients, moderately-differentiated in 164 (32.7%) patients, and poorly-differentiated tumors in remaining 328 (65.5%) patients. According to Borrmann type, 57 (10.4%) were type 0, 36 (7.2%) type I, 15 (3.0%) type II, 371 (74.0%) type III, 22 (4.4%) type IV. Of 501 patients, 159 (31.7%) had tumors located in the upper third, 72 (14.4%) had tumors in the middle third, 244 (48.7%) had tumors in the lower third of the stomach, and 26 (5.2%) had tumors occupied two-thirds or more of stomach. Lymph node metastasis was observed in 356 patients, the metastasis rate was 71.1%. The distribution of pathological stage was as follows: 35 (7.0%) patients belonged to stage IA, 52 (10.4%) IB, 60 (12.0%) IIA, 83 (16.5%) IIB, 88 (17.6%) IIIA, 83 (16.5%) IIIB, and 100 (20.0%) IIIC.

### p53 expression

**Figure 1:**
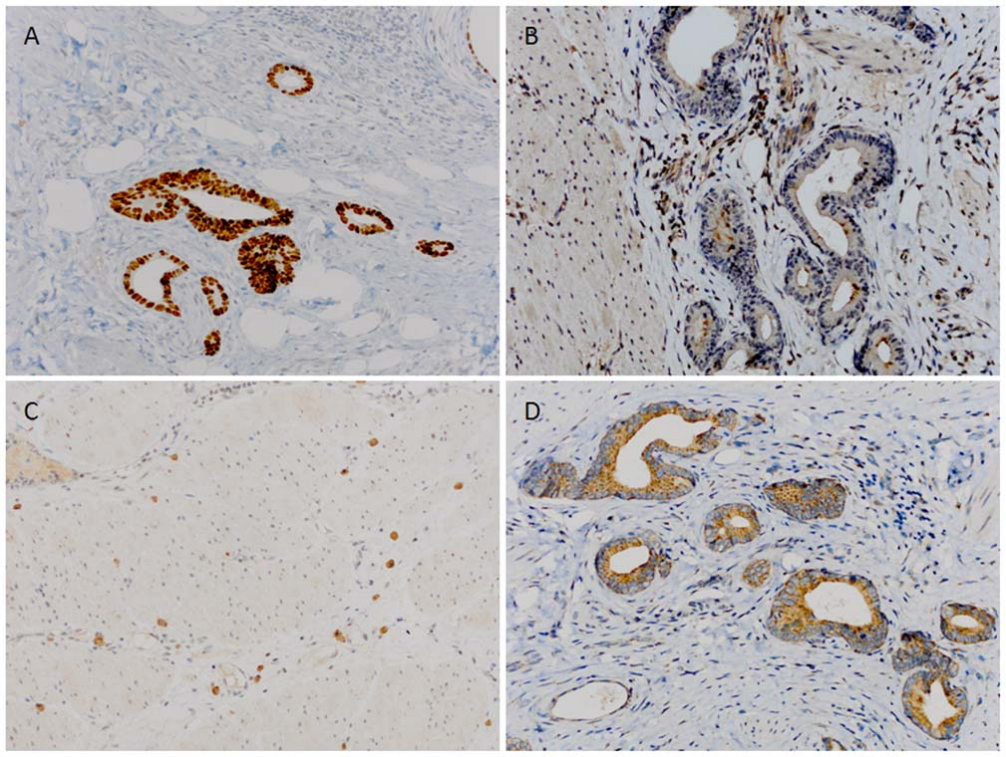
Positive expression of apoptosis-related biological markers immunohistochemistry in gastric cancer tissue. A) Positive expression of p53. B) Positive expression of bcl-2. C) Positive expression of bax. D) Positive expression of c-myc. (Original magnification: all ×100).

p53 expression was positive in 64.9% of all gastric cancer tissues. p53 staining was observed in the nucleus of carcinoma cells (Figure 1). p53 expression was associated with bax and c-myc (Table 1). Well-differentiated histology correlated with p53 expression. There was no correlation with gender, age, Borrmann type, tumor location, lymph node metastasis, and pathological stage (Table 2).

**Table 1:** Positive results of correlations among immunohistochemical markers.

Immunohistochemical markers		Correlation coefficients (r)	P value
p53	Bax	0.097	0.029
p53	c-myc	0.171	0.000
bcl-2	Bax	0.139	0.002
Bax	c-myc	0.132	0.003

**Table 2:** Positive results of correlations between immunohistochemical markers and clinicopathological parameters.

Variable		p53+		Bcl-2+		bax+		c-myc+	
	n	%	*P*	%	*P*	%	*P*	%	*P*
Sex			0.156		0.980		0.016		0.549
Female	162	60.5		22.2		35.2		56.2	
Male	339	66.9		22.1		46.6		58.9	
Age (y)			0.239		0.676		0.733		0.720
<60	284	62.7		21.5		42.3		57.4	
≥60	217	67.7		23.0		43.8		58.9	
Histological type			0.000		0.178		0.006		0.190
well-differentiated	9	88.8		0.00		44.4		66.6	
moderately-differentiated	164	72.6		25.0		53.0		63.4	
poorly-differentiated	328	60.3		21.3		37.8		55.2	
Tumor site			0.421		0.623		0.000		0.649
Upper third	159	66.7		23.8		55.9		59.7	
Middle Third	72	59.7		18.1		27.8		62.5	
Low third	244	63.9		22.9		39.3		55.3	
Two-thirds or more	26	76.9		15.4		38.5		61.5	
Borrmann type			0.454		0.332		0.002		0.002
0	57	56.1		14.0		19.3		33.3	
I	36	66.7		25.0		41.7		55.5	
II	15	60.0		33.3		53.3		60.0	
III	371	65.5		23.2		46.9		62.2	
IV	22	77.3		13.6		31.8		54.5	
Lymph nodes status			0.152		0.248		0.025		0.214
Yes	356	62.9		20.7		46.1		59.8	
No	145	69.6		25.5		35.2		53.8	
Pathological stage			0.561		0.000		0.026		0.243
I	87	60.9		18.4		29.8		51.7	
II	143	67.8		34.3		46.1		55.9	
III	271	64.6		16.9		45.4		61.3	

### bcl-2 expression

bcl-2 expression was positive in 22.2% of all gastric cancer tissues. bcl-2 staining was observed in the cytoplasm and cytomembrane of carcinoma cells (Figure 1). bcl-2 expression was associated with bax (Table 1). There was correlation with pathological stage, but no correlation with gender, age, histological type, Borrmann type, tumor location, lymph node metastasis, and (Table 2).

### bax expression

bax expression was positive in 42.9% of all gastric cancer tissues. bax staining was observed in the cytoplasm of carcinoma cells (Figure 1). bax expression was associated with p53, bcl-2 and c-myc (Table 1). Female gender, moderately-differentiated, Borrmann II, upper third location, and lymph node metastasis correlated with bax expression. As the pathological stage increased, the expression of bax became more frequent. There was no correlation with age (Table 2).

### c-myc expression

c-myc expression was positive in 58.1% of all gastric cancer tissues. c-myc staining was observed in the cytoplasm of carcinoma cells (Figure 1). c-myc expression was associated with p53, and bax (Table 1). Borrmann III correlated with c-myc expression. There was no correlation with gender, age, histological type, tumor location, lymph node metastasis, and pathological stage (Table 2). Additionally, raw data on clinicopathological features and immunoreactivity evaluations for 501 cases of gastric cancer was showed as a supplementary table in an Excel format (Table S1).

### Univariate analysis

**Figure 2:**
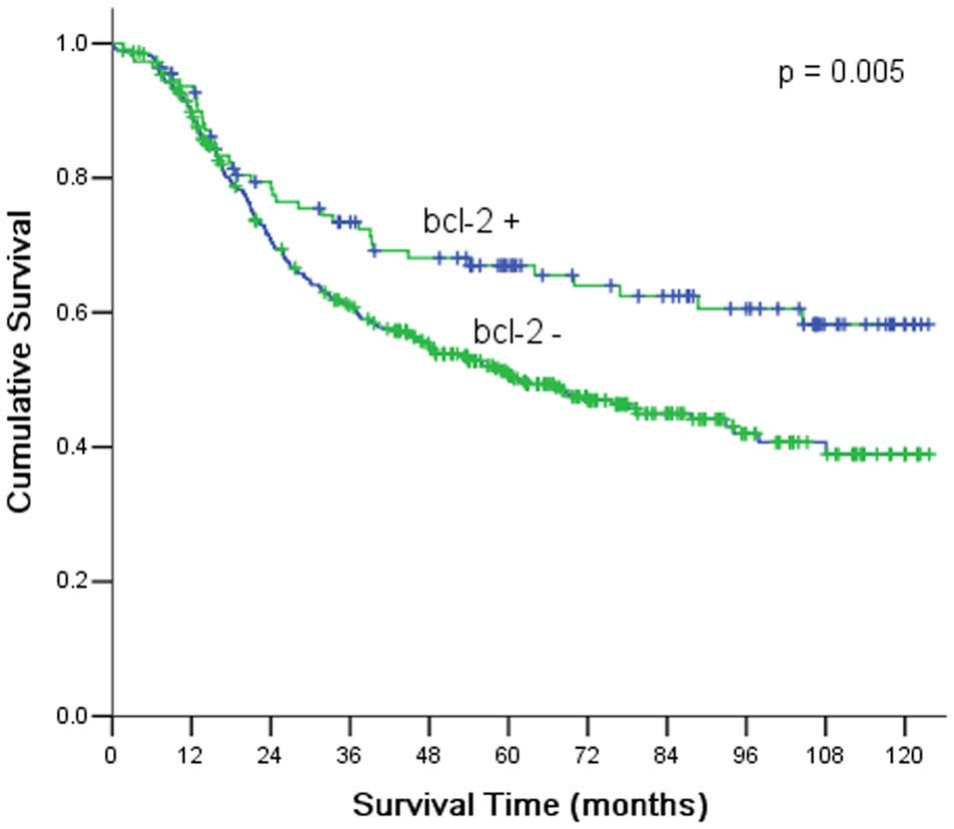
Kaplan-Meier survival curves according to the expression of bcl-2. There were significant differences between bcl-2+ and bcl-2− (*P* = 0.005).

The over-all five-year survival rate was 53.7% for all 501 patients. Five-year survival rate was 64.9% and 50.5% in bcl-2 positive and bcl-2 negative patients respectively, and this difference was statistically significant (Fig. 2). Except for bcl-2 expression, the significant prognostic factors included: tumor site, Borrmann type, status of lymph nodes, and pathological stage. Table 3 showed findings at univariate analysis for prognostic factors.

**Table 3:** Univariate analysis of all patients by Kaplan-Meier method.

Variable	n	5-Year survival rate (%)	Log rank χ^2^ value	P value
Sex			0.374	0.541
Female	162	56.8		
Male	339	52.2		
Age (y)			0.484	0.487
<60	284	56.0		
≥60	217	50.7		
Tumor site			31.896	0.000
Upper third	159	42.8		
Middle third	72	44.4		
Lower third	244	66.0		
Two-thirds or more	26	30.8		
Borrmann type			43.615	0.000
0	57	87.7		
I	36	44.4		
II	15	60.0		
III	371	50.9		
IV	22	22.7		
Lymph nodes metastasis			63.969	0.000
Yes	356	42.1		
No	145	82.1		
Pathological stage			106.030	0.000
I	87	93.1		
II	143	65.0		
III	271	35.1		
Bcl-2			7.712	0.005
+	111	64.9		
−	390	50.5		

### Multivariate analysis

Multivariate survival analysis, including all significant prognostic factors mentioned in univariate analysis, was performed to determine the independent prognostic factors for gastric cancer. Multivariate analysis using Cox proportional hazards model showed that bcl-2 expression and pathological stage were independent prognostic factors (Table 4).

**Table 4:** Multivariate analysis of patients by Cox model.

Variable	χ^2^	*P* vale	RR	95% CI
Sex	0.123	0.726	1.053	0.788–1.409
Age	2.257	0.133	0.816	0.625–1.064
Borrmann type	0.128	0.721	1.035	0.858–1.247
Lymph node metastasis	1.227	0.268	0.739	0.433–1.262
Tumor site	3.500	0.061	0.880	0.770–1.006
Pathological stage	45.611	0.000	1.517	1.344–1.712
Bcl-2	4.935	0.026	1.491	1.048–2.121

### Comparison of survival according to biological markers at same stage

**Figure 3:**
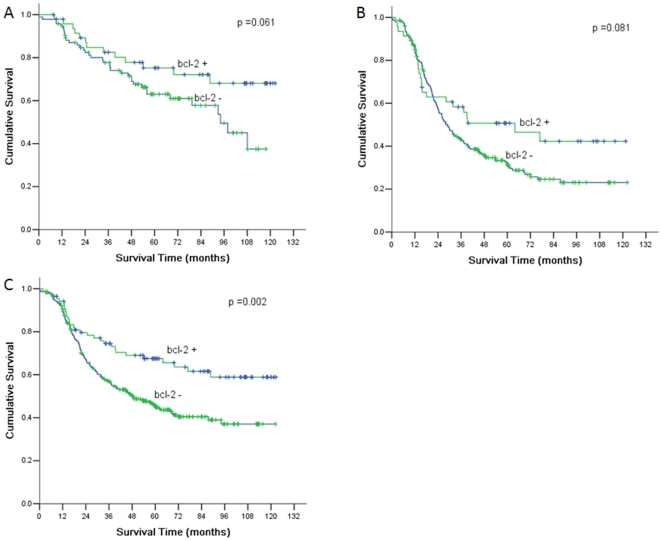
Comparison of survival according to Borrmann III and tumor stage. A) There were significant differences between bcl-2+ group and bcl-2− group according to Borrmann III (*P* = 0.002). B) There were no significant differences according to stage II. C) There were no significant differences according to stage III.

According to 7th UICC TNM Staging System, gastric cancer was divided into 7 stages: stage IA, stage IB, stage IIA, IIB, IIIA, IIIB, IIIC. Basing on the bcl-2 expression, each stage was divided into bcl-2 positive and bcl-2 negative group. There were significant differences of over-all 5-year survival between bcl-2 positive and bcl-2 negative group according to stage IIB (*P* = 0.003). Additionally, we found that there were significant differences of over-all 5-year survival between bcl-2 positive and bcl-2 negative group according to Borrmann III (*P* = 0.002); no significant differences according to stage II and stage III (Figure 3).

## Discussion

The identification of prognostic factors in gastric cancer was essential for predicting patients' survival and determining optimal therapeutic strategies. Many studies have indicated that the depth of invasion and lymph node metastasis were the most important prognostic factors in gastric cancer [17], [18]. As a result of the variability of prognosis within same pathological stage of gastric cancer, there have been a lot of researches for specific biological markers to identify patients with poorer prognosis [19]–[21]. Meanwhile, the expression of p53, bcl-2, bax, and c-myc were known for being related with tumorogenesis. Therefore, the aim of this study was to investigate whether these biological markers can be used as additional prognostic factors to traditional TNM stage in gastric cancer. In this study, the expression of these proteins was investigated in 501 cases of resected primary human gastric cancers.

The immunohistochemical expression rates of p53, bcl-2, bax, and c-myc were 64.9%, 22.2%, 42.9%, 58.1%, respectively, and these results were consistent with the expression rates of other studies reported as 17–84.1%, 12.7–67%, 42.4–58.0%, 23.5–72.9%, respectively [9], [12], [22]–[26]. p53 played an important role in apoptosis. It altered most frequently in human cancer. Wild-type p53 protein induced growth arrest at the G1/S phase of cell cycle in response to DNA damage, thus preventing the proliferation of cells. Muted-type p53 lost the function, and allowed cells with damaged DNA to proliferate. In this study, a reverse correlation between p53 and histological type was found, which demonstrated that deregulation of p53 might result in uncontrolled proliferation in gastric cancer. It was consistent with a previous study [22]. It was controversy as for the prognostic value of p53, some studies have reported that p53 was associated with poor prognosis [27], [28], while others reported that p53 has no influential on prognosis [29], [30]. Our research showed that p53 was not related to the prognosis of gastric cancer. bax acted as an accelerator of apoptosis on cell life. A previous study proposed that the ratio of bax to bcl-2 played a critical role in regulating the propensity for apoptosis [31]. In the present study, the expression of bax was associated with that of bcl-2, and bax overexpression correlated significantly with some clinicopathological features such as gender, histological type, Borrmann type, tumor location, and lymph node metastasis. We found that the patients with positive expression of bax were prone to present lymph node metastasis, and bax was not an independent prognostic factor. This result was different from a previous research. Anagnostopoulos et al. [24] reported that negative bax expression in gastric was associated with lymph node metastasis and poor clinical prognosis. The reasons for the conflicting results were not clear. It was possible that the gene differences between western people and eastern people contributed to this. The proto-oncogene c-myc was a master regulator of cell proliferation and transformation through both transcriptional and nontranscriptional means, and was frequently deregulated in human malignancies. Ninomiya et al. [26] reported that excess reactivity to c-myc product occurred more frequently in invasive cancers than in localized cancers, and c-myc production expression in cancer tissue correlated well with peritoneal dissemination, and patients with c-myc positive expression had poorer prognosis than those with c-myc negative expression. In present study, we did not find any relationship between c-myc expression and invasive behaviour and prognosis other than Borrmann type. We found that c-myc expression was more associated with Borrmann III.

The bcl-2 gene was firstly described as an oncogene in follicular lymphoma. It was well known to inhibit apoptosis [32], but some studies have reported that overexpression of bcl-2 suppressed cellular proliferation and was associated with less aggressive biological behaviour [33], [34]. bcl-2 has been reported in a variety of human epithelial malignant tumors including gastric carcinoma. However, the exact role and clinical significance of bcl-2 remained to be elucidated. Previous studies have shown that the expression of bcl-2 was associated with better prognosis in non-small-cell lung cancer and gastric cancer [35], [36]. Pietenpol et al. [37] reported that overexpression of bcl-2 inhibited the growth of several solid tumor cell lines. Saegusa et al. [33] demonstrated that the majority of bcl-2+ cancer cells was in a nonproliferative state, and the average expression of Ki-67 labeling index and apoptotic labelling index in bcl-2+ foci were significantly lower than that in bcl-2- foci. Aizawa et al. [36] showed that the expression of bcl-2 in advanced gastric cancer was associated with a lower apoptotic index and a better prognosis. All these studies suggested that bcl-2 not only inhibited apoptosis, but also suppressed cellular proliferative activity. In present study, we characterized for the first time the expression of bcl-2 proteins in Chinese gastric cancer patients. Our data showed that bcl-2 was significantly correlated with better survival.

The identification of prognostic factors in gastric cancer was essential for predicting patients' survival and determining optimal therapeutic strategies. It was well known that the depth of invasion and the presence or absence of lymph node metastasis were the most important prognostic factors in gastric cancer. However, it was impossible to precisely predict the prognosis through the conventional staging procedures as a result of the variability of prognosis within the same stage of gastric cancer. Liu et al. [5] found that there were significant differences between the larger-size tumor group and the small-size tumor group according to stage IIIb and IV. Therefore, it was essential to search for the other specific factors to identify subgroups of patients with more aggressive course of the disease especially in the 7th editions of the UICC TNM Staging System. According to the 7th UICC TNM classification, each stage of gastric cancer was further divided into bcl-2 positive and bcl-2 negative group. Our data showed that the expression of bcl-2 greatly influenced prognosis in stage IIB. The results indicated that the expression of bcl-2 could provide additional prognostic information in the patients with gastric cancer.

Some limitations of this study should be acknowledged. Firstly, the status of Helicobacter pylori was not described. As a crucial carcinogen of gastric cancer, Helicobacter pylori could be linked to some other parameters such as bcl-2. Secondly, the relationship between tumor recurrence and bcl-2 was not investigated.

In conclusion, the expression of bcl-2 can serve as one useful indicator for predicting the prognosis of patients with gastric cancer. Therefore, it might be a candidate for the staging system in addition to conventional factors.

## Supporting Information

Table S1 Raw data on clinicopathological features and immunoreactivity evaluations for 501 cases of gastric cancer.
(XLS)
